# Contre-Coup

**Published:** 1907-03-16

**Authors:** 


### Points in Surgery.

### CONTRE-COUP.

It is well known that violence applied to the cranium may not only affect the soft structures immediately under the bone at the site of the direct injury, but may also cause considerable damage to that part of the brain which is diagonally opposite to the place where the blow or other immediate violence is inflicted. For instance, a severe blow on the lateral aspect of the right side of the head may be followed by a right-sided hemiplegia, indicating that the left side of the brain, though not itself directly struck, has been much damaged. This effect produced upon the brain on the side directly opposite to the point of trauma is known as injury by contre-coup. In such a case the question at once arises: Where should one trephine? over the site of direct injury, or over the opposite side? Or should one trephine over both sides?

The answer to this question can only be given when one knows the morbid anatomy of such cases. Trephining in such circumstances would be performed, presumably, for one or more of three purposes, namely: (1) The elevation of depressed bone; (2) the ligature of a bleeding vessel; (3) the evacuation from the meninges of blood-clot, which was compressing the brain.

Are any of these conditions likely to be present at the site of injury by contre-coup? The answer is: No, none of the three is likely. It is true that an injury to the top of the head, either by a direct blow from a hammer, for example, or by a fall on the head from a height, may cause a fracture of the base of the skull. This fractured base will be at the opposite side of the cranium to the point actually struck, and may appear to be due to contre-coup; but in reality it is not so much a pure contre-coup as a direct injury to the base. When, in falling from a height, the vertex of the skull strikes the ground, the body is still descending with considerable momentum; this momentum is suddenly checked, the whole force of it falling upon the base of the skull; fracture of the latter is thus due rather to direct injury from the momentum of the body than it is to contre-coup from the injury to the vertex. In any event, operation for fractured base of the skull is not practicable.

In true injury by contre-coup, what are the morbid changes likely to be found? On the side opposite to the actual injury it is very unusual to find any fracture of the bones, unless by direct extension of a fracture starting from the point struck. The maximum injury to the bone, apart from a fracture of the base, will be at the site of direct violence. Is meningeal hæmorrhage likely to occur at the point of contre-coup? Small hæmorrhages may be found, but it is extremely rare to find a large extravasation of blood capable of relief by operation, though this may of course exist on the same side as the immediate injury. What, then, are the changes produced by the contre-coup? They are nearly always lesions of the brain-substance, for the most part associated with local softening, and either with or without small cortical hæmorrhages which may extend for a variable depth into the cerebral tissue. How this softening is produced is uncertain; perhaps it is caused by the impact of the brain against the rigid cranial walls, perhaps in other ways; but the post-mortem evidence so constantly shows that the damage done by contre-coup is in the form of this softening, and is not due either to fractured bone or to a large blood-clot, that even if the cranial injury be associated with hemiplegia of the side opposite to the suspected contre-coup, trephining over the site of contre-coup is seldom, if ever, likely to be able to afford relief. In other words, if trephining is to be undertaken on account of a cranial injury, it should be upon the side of the direct injury, and not on the side on which there is clinical evidence of a lesion by contre-coup. If there is no indication to trephine at the site of the direct injury, there is probably noting to be gained by trephining at all. The contused brain tissue at the site of contre-coup cannot be cured by surgical means; it may, however, possibly recover by itself.

